# Proteomic analyses of urinary exosomes identify novel potential biomarkers for early diagnosis of sickle cell nephropathy, a sex-based study

**DOI:** 10.3389/fphys.2024.1300667

**Published:** 2024-02-15

**Authors:** Balamurugan Packialakshmi, Emily Limerick, Hans C. Ackerman, Xionghao Lin, Sergei Nekhai, James D. Oliver, Ian J. Stewart, Mark A. Knepper, Courtney Fitzhugh, Xiaoming Zhou

**Affiliations:** ^1^ Department of Medicine, Uniformed Services University of Health Sciences, Bethesda, MD, United States; ^2^ Cellular and Molecular Therapeutic Branch, National Heart Lung and Blood Institute, Bethesda, MD, United States; ^3^ Physiology Unit, Laboratory of Malaria and Vector Research, National Institute of Allergy and Infectious Diseases, Rockville, MD, United States; ^4^ Department of Medicine, Howard University, Washington, DC, United States; ^5^ Nephrology Service, Walter Reed National Military Medical Center, Bethesda, MD, United States; ^6^ System Biology Center, National Heart Lung and Blood Institute, Bethesda, MD, United States

**Keywords:** heparanase, cathepsin C, α2-macroglobulin, sarcoplasmic endoplasmic Ca^2+^ ATPase-3, albuminuria, sex difference, chronic kidney disease, gender difference

## Abstract

Sickle cell nephropathy (SCN) is a leading cause of morbidity and mortality in sickle cell disease (SCD). Early intervention is crucial for mitigating its effects. However, current diagnostic methods rely on generic tests and may not detect SCN until irreversible renal damage occurs. Therefore, specific biomarkers for early diagnosis of SCN are needed. Urinary exosomes, membrane-bound vesicles secreted by renal podocytes and epithelial cells, contain both common and cell type-specific membrane and cytosolic proteins, reflecting the physiologic and pathophysiologic states of the kidney. Using proteomics, we analyzed the proteomes of urinary exosomes from humanized SCD mice at 2 months (without albuminuria) and 4 months (with albuminuria) of age. Excretion of 164 proteins were significantly increased and 176 proteins was significantly decreased in the exosomes when mice developed albuminuria. Based on the relevance to SCD, chronic kidney disease and Western blot confirmation in mice, we analyzed protein abundance of heparanase, cathepsin C, α2-macroglobulin and sarcoplasmic endoplasmic Ca^2+^ ATPase-3 (SERCA3) in the urinary exosomes and urine of 18 SCD subjects without albuminuria and 12 subjects with albuminuria using Western blot analyses. Both male and female subjects increased or tended to increase the excretion of these proteins in their urinary exosomes upon developing albuminuria, but female subjects demonstrated stronger correlations between the excretion of these proteins and urine albumin creatinine ratio (UACR) compared to male subjects. In contrast, exosomal excretion of Tamm-Horsfall protein, β-actin and SHP-1 was independent of albuminuria. These findings provide a foundation for a time-course study to determine whether increases in the levels of these proteins precede the onset of albuminuria in patients, which will help determine the potential of these proteins as biomarkers for early detection of SCN.

## Introduction

Sickle cell disease (SCD) is a hereditary blood disorder characterized by a mutation of glutamic acid to valine in both chains of β-globin. This genetic alteration leads to the pathologic polymerization of hemoglobin, resulting in the deformation of red blood cells. As a consequence, these cells become rigid and encounter difficulty passing through narrow blood vessels. This process leads to various complications in renal tissues, including ischemia, vasoconstriction, infarction, inflammation, and the activation of platelets and coagulation (Becker, 2011; Nath and Hebbel, 2015; Ataga et al., 2022). Approximately 40% of SCD patients develop sickle cell nephropathy (SCN) (Becker, 2011). In these cases, SCD significantly alters kidney structure and disrupts nearly all major renal physiological processes (Becker, 2011; Nath and Hebbel, 2015; Ataga et al., 2022). Moreover, approximately 4%–18% of SCD patients progress to end-stage kidney disease (ESKD), necessitating treatments such as dialysis or kidney transplantation (Becker, 2011). Unfortunately, the average survival rate following the onset of ESKD is only 4 years, and a substantial 40% of SCD patients on dialysis succumb within 20 months (Becker, 2011).

Sickle cell nephropathy is a progressive condition. Patients with SCD develop urinary concentration defects, increased glomerular filtration rate (GFR) and hematuria as early as infancy. With increasing age, some patients may develop micro-albuminuria and then macro-albuminuria, leading to ESKD (Becker, 2011; Nath and Hebbel, 2015; Wang et al., 2019). In the progression of SCN, a sex difference is observed. Both adult and pediatric male patients exhibit a more rapid decline in estimated GFR compared to their female counterparts (Kasztan et al., 2020; Ataga et al., 2023). Early interventions may help prevent or mitigate SCN, as demonstrated by successful early interventions for diabetic nephropathy (Molitch et al., 2004). The current diagnosis of SCN relies on tests, such as serum creatinine levels and urine albumin excretion, which may manifest too late for optimal interventions and management (Voskaridou et al., 2006; Sundaram et al., 2011). To address this problem, researchers have explored novel biomarkers in the blood and urine for early detection of SCN. Some of the potential biomarkers reported include kidney injury molecule-1 (KIM-1), N-acetyl-β-D-glucosaminidase, endothelin-1, TGF-β1, soluble urokinase-type plasminogen activator receptor, urinary macrophage stimulating protein, plasma and urinary orosomucoid, and ceruloplasmin (Voskaridou, Terpos, Michail, Hantzi, Anagnostopoulos, Margeli, Simirloglou, Loukopoulos and Papassotiriou, 2006; Mohtat et al., 2011; Sundaram, Bennett, Wilhelm, Kim, Atweh, Devarajan and Malik, 2011; Jerebtsova et al., 2018; Jerebtsova et al., 2020; Nekhai et al., 2021; Afangbedji et al., 2022). However, questions remain regarding the specificity and reproducibility of some of these biomarkers (Mohtat, Thomas, Du, Boakye, Moulton, Driscoll and Woroniecki, 2011; Sundaram, Bennett, Wilhelm, Kim, Atweh, Devarajan and Malik, 2011; Hamideh et al., 2014). Some of these biomarker candidates are non-specific and may increase in urine or serum as a result of damage in other organs without known renal injury (Tsai et al., 1997; Finazzi et al., 2001; Holzscheiter et al., 2014; Wasung et al., 2015). Additionally, whether sex differences affect these biomarkers remains largely unknown.

Urinary exosomes are membrane-bound vesicles secreted by renal podocytes and epithelial cells facing the urine and urinary drainage system. They contain both common and cell type-specific membrane and cytosolic proteins, providing valuable insights into the physiological and pathophysiological states of the kidney (van Balkom et al., 2011). Urinary exosomes have shown promise as a source of specific biomarkers for renal diseases (Hoorn et al., 2005). Therefore, our hypothesis was that unique protein fingerprints in urinary exosomes could predict the risk of SCN and potentially serve as biomarkers for early SCN diagnosis. To test this hypothesis, we initially compared the proteomes of urinary exosomes from the same humanized SCD mice (Townes model) when they had no albuminuria with when they developed albuminuria. We identified differentially increased proteins. Subsequently, we examined whether some of these findings could be reproduced in SCD patients, using Western blot analyses. Because sex plays a critical role in SCN, we analyzed the data separately based on sex. Our results show that when albuminuria developed, both men and women showed an increase or a tendency to increase in the release of heparanase, cathepsin C, α2-macroglobulin, and sarcoplasmic endoplasmic Ca2+ ATPase-3 (SERCA3) in their urinary exosomes. However, in women, there was a stronger correlation between the release of these proteins and the urine albumin creatinine ratio (UACR) compared to men. This data forms the basis for future studies, which will investigate if the elevation of these protein levels occurs before the onset of albuminuria.

## Materials and methods

### Animals

All animal procedures were approved by the Institutional Animal Care and Use Committee of the Uniformed Services University of the Health Sciences (Protocol # MED-16-978). Townes SCD mice and their heterozygous (non-SCD) controls (JAX Stock: 013071) as well as C57BL/6 mice were purchased from The Jackson Laboratory and housed in the university vivarium on a 12:12 light:dark cycle with *ad libitum* access to regular food and water. To avoid sex bias, both male and female mice were used. Urine was collected from mice housed in metabolic cages using methods previously described (Zhou et al., 2014), and each collection vial contained a quarter of a Roche protease inhibitor cocktail tablet to prevent protein degradation. Urine samples were centrifuged at 2,000 g for 10 min at 4°C to remove debris and stored at −80°C until further analysis.

### Glomerular filtration rate and urine albumin measurements

Mouse transcutaneous glomerular filtration rate (tGFR) was measured as previously described (Packialakshmi et al., 2022). Briefly, a transdermal NIC-Kidney unit with internal memory (Mannheim Pharma and Diagnostics GmbH) was mounted on the back of a mouse. Fluorescein isothiocyanate (FITC)-sinistrin (15 mg/100 g BW; dissolved in saline at 35 mg/mL, Mannheim Pharma and Diagnostics GmbH) was injected via the intraocular route 2 min after the mounting. The mouse was placed in a single cage for an hour for recording and the data was analyzed with the MPD 1.0 software (Scarfe et al., 2018). The t^1/2^ value was used to calculate the tGFR. Mouse urine albumin levels were measured using the Mouse Albumin Assay Max ELISA kit (catalog # EMA3201-1 from AssayPro) according to the manufacturer’s protocol.

### Histology

The renal structure of humanized SCD mice was analyzed with Hematoxylin-eosin (H & E) and Masson’s trichrome (MTC) staining.

### Mouse urine exosomes isolation and digestion

**FIGURE 1:**
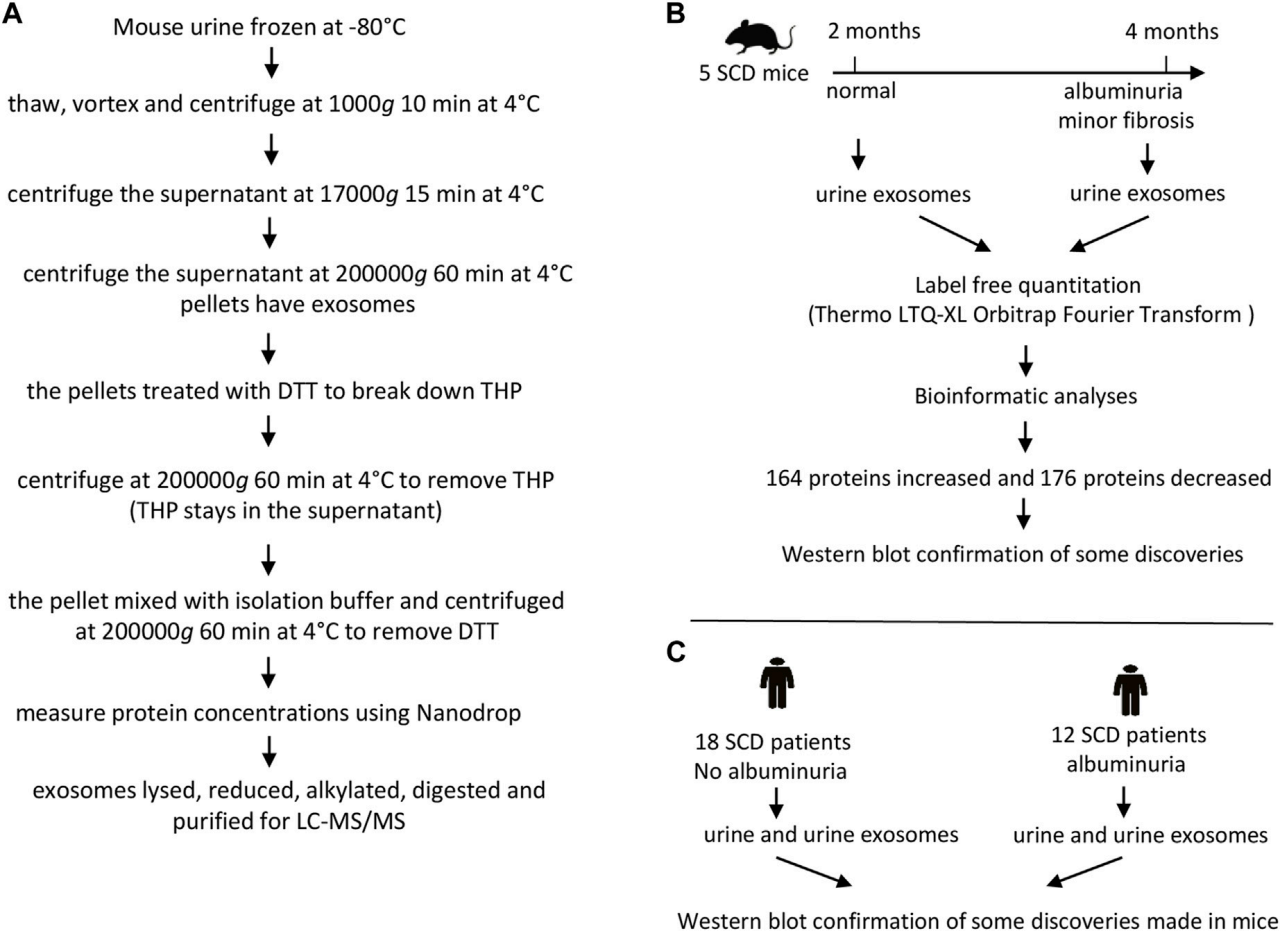
The flow charts of experimental protocol and designs. **(A)** Protocol for preparing mouse urinary exosomes for liquid chromatography-tandem mass spectrometry (LC-MS/MS). **(B)** Design for proteomic analyses of mouse urinary exosomes. **(C)** Design for Western blot analyses of human urine and urinary exosomes.

The exosomes were extracted from the urine of 5 SCD mice (2 males and 3 females) when they were 2 and 4 months old respectively. The urine (∼4 mL pooled from consecutive collections) from each mouse was thawed, mixed and followed by centrifugation at 1,000 g for 10 min at 4°C and the supernatant was again centrifuged at 17,000 g for 15 min at 4°C. The supernatant was then subjected to ultracentrifugation at 200,000 g for 60 min at 4°C to precipitate pellets. The pellets containing exosomes were treated with freshly prepared 200 mg/mL dithriothretol (DTT) in an isolation buffer (10 mM triethanolamine and 250 mM sucrose, pH 7.6 with the Roche protease inhibitor tablets) and heated for 2 min at 95°C to break down Tamm-Horsfall protein also known as uromodulin. The solution was centrifuged at 200,000 g for 60 min at 4°C. The pellets were dissolved again in the isolation buffer and centrifuged at 200,000 g for 60 min at 4°C to remove DTT (Zhou et al., 2006b). The final pellets were dissolved in ∼400 μL of the isolation buffer. The exosomes were examined under an electron microscope. The protein contents of the exosomes were measured at A280 nm with a NanoDrop (ThermoFisher). The exosome samples (400 µg) were dried under speed vacuum with a DNA110 Speed Vac under no heating (Forma Scientific) and then dissolved in 6 M urea buffer (6 M urea and 50 mM Tris-HCl, pH 8.0). The samples were treated with 10 mM DTT for 60 min at 60°C and alkylated with 40 mM freshly prepared iodoacetamide (IAA). Excess IAA was neutralized with 10 mM DTT, and the samples were digested with 40 ng/μL trypsin (Pierce) at 37°C for 48 h. The digested peptides were dried and purified with C18 spin columns (Pierce) (Figure 1A).

### Electron microscopy

5–10 µL of sample were applied to a standard 3 mm formvar-carbon coated grid (Electron Microscopy Sciences, Hatfield, PA) for 5 min and the excess was wicked off with a piece of filter paper. The grid was then washed very briefly on 3 drops of water to remove buffer salts. Following this, 5 µL of 2% aqueous uranyl acetate was applied to the grid for 1 min before the excess was again wicked off. The grid was then allowed to air dry for several minutes before examination in a JEOL JEM-1011 TEM (JEOL United States Inc., Peabody, MA). Images were captured using an AMT XR50S-A digital camera (Advanced Microscopy Techniques, Woburn, MA).

### Liquid chromatography-tandem mass spectrometry (LC-MS/MS) and bioinformatics

The purified peptides were dried and dissolved in 50 μL of 0.1% formic acid. Aliquots of 10 μL tryptic peptides were loaded to a LC-20AD Nano HPLC system (Shimadzu Corporation, Columbia, MD, United States) coupled to LTQ XL Orbitrap mass spectrometer (Thermo Fisher Scientific) with the installed Xcalibur software (version 2.0.7, Thermo Fisher Scientific). Liquid chromatography was carried out on an in-house made Nano-HPLC column (Polymicro Technologies Inc., Phoenix, AZ, United States) packed with reverse phase PolySulfoethyl A, 5 µM, 200 Å (PolyLC Inc., Columbia, MD, United States). Full-scan mass spectra were acquired in the Orbitrap over 300–2,000 *m/z* with a resolution of 30,000. Three most intense ions were selected for fragmentation using collision-induced dissociation (CID). Samples from each mouse were run in triplicate. Protein identifications were carried out using Proteome Discoverer 2.3 software in combination with the SEQUEST protein database search engine. A sequential database search was performed using the Uniprot mouse database (1/23/2019, 4195 sequences) at a false discovery cut off ≤1%. Label-free quantitation was performed and the results were exported as *.xls files for analysis. Label-free quantitation was performed. The list of differentially expressed proteins were shortlisted based on *p*-value <0.05, unique peptide ≥1, reproducibility (must be present in all the 10 samples and their 3 replicates) and identified with medium or high confidence. The protein results were analyzed by DAVID GO for their known functions (Huang et al., 2009) (Figure 1B). A Venn Diagram was created using a free on-line program (https://venngage.com) (Figure 3B). A Vocano plot was generated with R program (https://www.r-project.org). Log2 was used as a predetermined threshold for the fold change (FC) (Figure 3C).

### Participant enrollment and urine collection

All procedures were approved by the Institutional Review Board of National Heart Lung and Blood Institute (Clinical Trials identifier: NCT03958643). Adults with sickle cell anemia (HbSS or HbSβ^0^-thalassemia) were eligible to participate. Participants were recruited from 24 May 2019 to 1 May 2021. All participants provided written informed consent. Study procedures were performed at subject’s clinical baseline. Participants on stable hydroxyurea, antihypertensive medication, and/or chronic transfusion therapy were allowed to enroll. The estimated glomerular filtration rate (eGFR) was calculated based on serum cystatin C and creatinine (2021 equation). Both cystatin C and creatinine were measured in a clinical lab with cystatin C measured in a Cobas C (Roche) and creatinine measured in an Architect 39 (Abbott). Patients eGFR <60 mL/min/1.73 m^2^, HIV, hepatitis B or C, chronic inflammatory condition, acute illness, uncontrolled hypertension, and pain crisis within 4 weeks were excluded. Subjects with nephropathy were identified by albuminuria (≥30 mg/g). Among these subjects, two had an eGFR ranging from 60 to 70 mL/min/1.73 m^2^, while the remaining subjects had an eGFR greater than 90 mL/min/1.73 m^2^. The second and third morning urine samples were collected from outpatient clinic patients who were then divided into those with (≥30 mg/g) and without (<30 mg/g) albuminuria (Table 1). A Roche protease inhibitor cocktail tablet was added to the urine collection container to prevent protein degradation. A total of 31 subjects were recruited, but the urine sample from one subject was not used due to technical reasons. Therefore, 18 participants without albuminuria and 12 subjects with albuminuria were included in the study (Table 1).

**TABLE 1:** Subjects’ profiles.

	SCD subjects with no albuminuria	SCD subjects with albuminuria
Sample size	18	12
Male: Female	10:8	5:7
Median age (range)	32.5 (18–45)	30 (24–55)
Race	Black or African American	Black or African American (1 unknown)
Ethnicity	Not Latino or Hispanic (1 unknown)	Not Latino or Hispanic (1 unknown)
Mean Urinary Albumin Creatinine Ratio (mg/g)	7.0 ± 2.1	389.5 ± 169.2*
Mean eGFR CKD-EPI Creatinine Equation (2021) (ml/min/1.73 m2)	125.4 ± 3.2	107.4 ± 7.2*

*p < 0.05, unpaired *t*-test.

### Participants’ urine exosomes isolation for Western blot analyses

The urine (∼25 mL) from each subject was thawed, mixed, and centrifuged at 1,000 g for 10 min at 4°C. The supernatant was again centrifuged at 17,000 g for 15 min at 4°C. The supernatant was then subjected to ultracentrifugation at 200,000 g for 60 min at 4°C to precipitate exosomes. The exosomes were dissolved in PBS buffer with protease inhibitor tablets and examined under an electron microscope. The protein concentrations of exosomes were estimated using the BCA method and dissolved in 4X SDS loading buffer (Figure 1C). Urine samples were directly used without any extraction. The protein concentrations in the urine were estimated using the BCA method and then dissolved in 4X SDS loading buffer as well (Figure 1C).

### Western blot analysis

Equivalent amount of protein samples in the SDS loading buffer (mouse exosomes = 12 µg/lane, human urine exosomes = 20 μg/lane, human urine = 50 µg/lane) were fractionated in a 4%–12% Bis-Tris gel (ThermoFisher). Proteins in the gel were transferred to a nitrocellulose membrane and the membrane was submerged in the Odyessy blocking buffer (Li-Cor) or 5% non-fat milk in PBS for 60 min at room temperature. The membrane was probed with a primary antibody against heparanase (1:1000 dilution, Proteintech 24529-1-AP), cathepsin c (1:1000 dilution, ThermoFisher PA5-37849), α2-macroglobulin (1:1000 dilution, Proteintech 66126-1-Ig), SERCA3 (1:500 dilution, Proteintech 13619-1-AP), integrin αV (1:500 dilution, Proteintech 27096-1-1AP), podocalyxin (1:500 dilution, Proteintech 18150-1-AP), HSP27 (1:1000 dilution, Cell Signaling 2402), β-actin (1:1000 dilution Cell Signaling 3700), SHP-1 (1:500 dilution, SC-287) or Tamm-Horsfall protein (1:2500 dilution, SC-271022) at 4°C overnight. The membrane was then washed briefly and probed with a corresponding Alexa fluorophore conjugated secondary antibody at room temperature for an hour. An infrared imaging scanner (Li-Cor) was used to images and analyze protein abundance.

### Statistics

All data were presented as mean ± standard error (SE). Band intensity data were analyzed using an unpaired two-tailed *t*-test with statistical significance set at *p* < 0.05. Correlation analyses were performed using Pearson’s correlation test with GraphPad Prism 10.1.2.

## Results

### SCD mice increase urinary excretion of albumin at 4 months old with only minor fibrosis in the renal cortex

**FIGURE 2:**
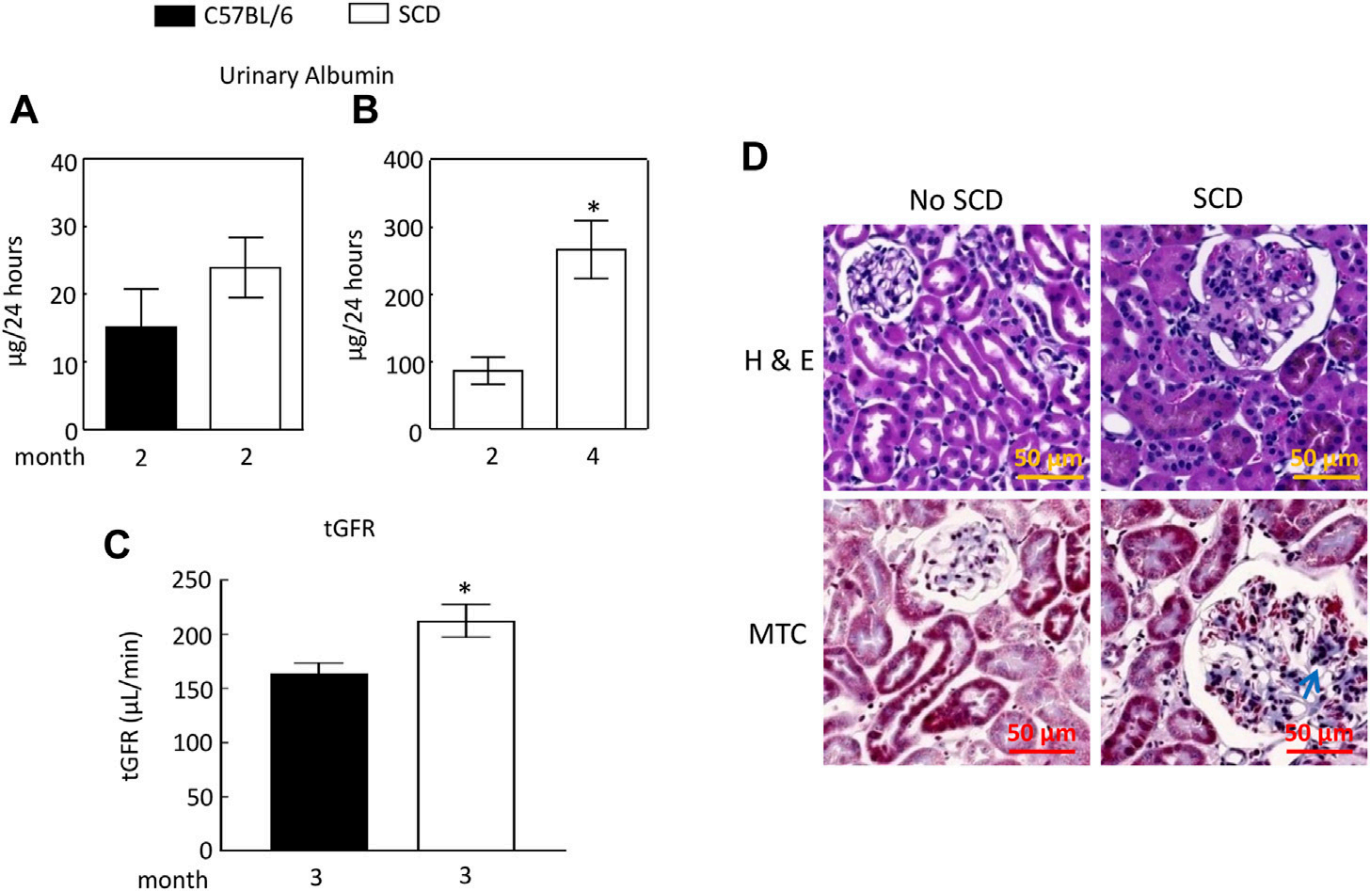
Sickle cell disease (SCD) mice have albuminuria when they reach 4 months old. **(A)** At 2 months old, there was no significant difference in urinary albumin excretion between SCD and C57BL/6 mice (*n* = 3, 2 males and 1 female in each strain). **(B)** By the age of 4 months, SCD mice exhibited a more than threefold increase in urinary albumin excretion compared to their levels at 2 months old (*p < 0.05, *n* = 5, 2 males and 3 females). It is worth noting that the disparities in urinary albumin levels observed between **(A,B)** for 2-month-old SCD mice are likely attributable to variations in measurement times and the use of different sets of SCD mice for each measurement. Urinary albumin levels were quantified using an ELISA kit (EMA3201, AssayPro). **(C)** SCD mice showed significantly elevated transcutaneous glomerular filtration rate (tGFR) compared to C57BL/6 mice (*p < 0.05, *t*-test, *n* = 5, 2 males and 3 females). The tGFR was determined by measuring the disappearance of fluorescein isothiocyanate (FITC)-sinistrin from the mouse body. **(D)** At 4 months old, SCD mice displayed enlarged glomeruli and minor fibrosis (indicated by arrows) compared to non-SCD mice (heterozygotes), as shown in representatives from three independent experiments. H & E, hematoxylin-eosin staining; MTC, Masson’s trichrome staining.

We found that urinary excretion of albumin in SCD mice (2 males and 1 females) at 2 months old and was not significantly different from that of C57BL/6 mice (2 males and 1 females) (Figure 2A). We then used a different cohort of SCD mice (2 males and 3 females) and collected urine from each SCD mouse at this age, whose urinary excretion of albumin served as the baseline. We monitored the SCD mice urinary excretion of albumin every two to 3 weeks and found that SCD mice urinary albumin excretion rate was more than tripled when mice reached 4 months age (87 ± 20 vs. 267 ± 43 µg/24, Figure 2B). The SCD mice had a significantly higher tGFR than C57BL/6 mice (212 ± 15 vs. 164 ± 10 μL/min, Figure 2C). Histology analyses revealed that SCD mice had enlarged glomeruli compared with heterozygotes (non-SCD), a typical feature observed in both SCD mice and patients, but only had minor fibrosis when they reached 4 months old (Figure 2D).

### Proteomic analyses of the mouse urinary exosomes

**FIGURE 3:**
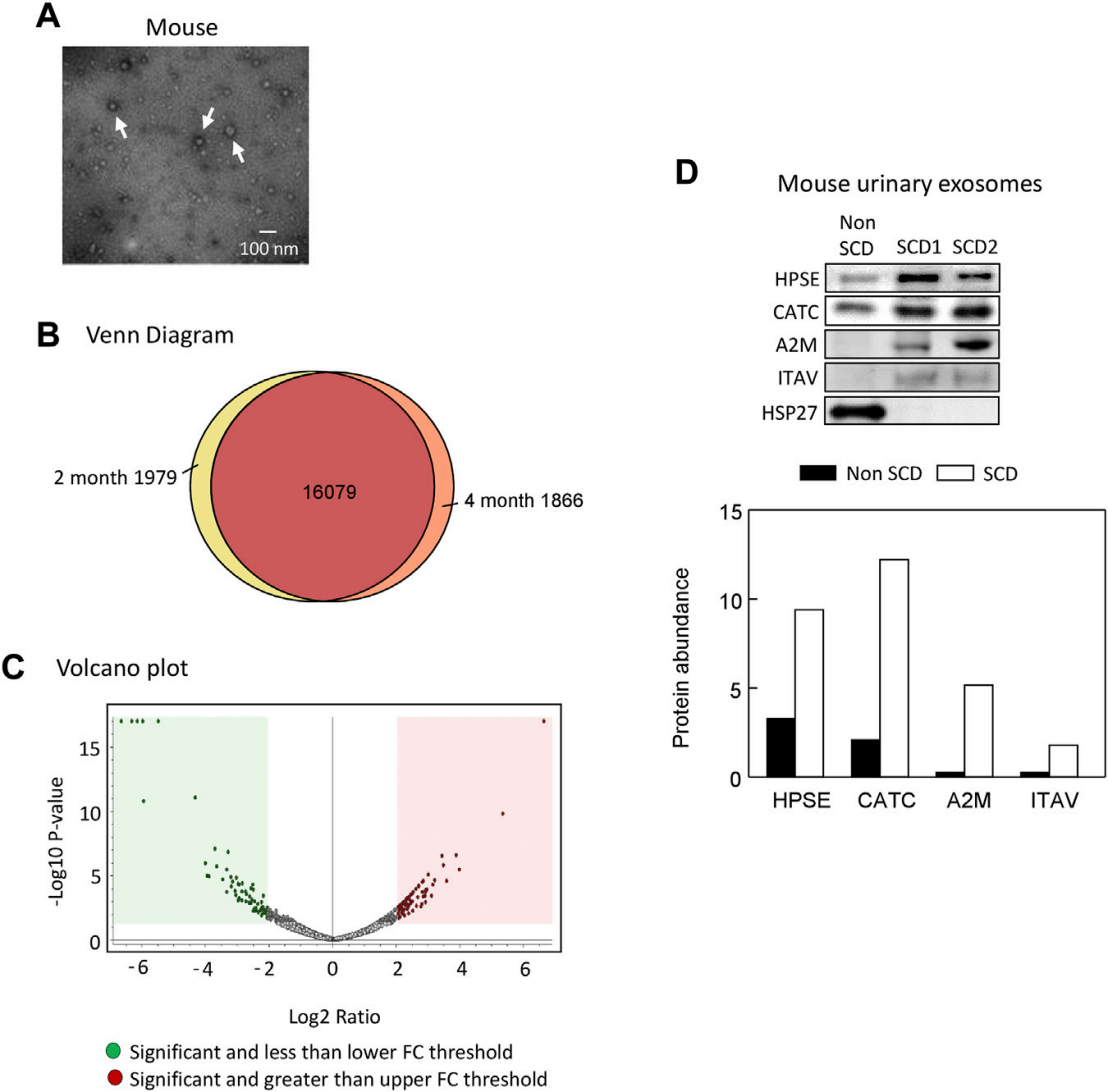
Proteomic analyses of mouse urinary exosomes. **(A)** Confirmation of urinary exosome morphology by electron microscopy. Urinary exosomes were extracted through differential centrifugation and verified using an electron microscope (JEOL JEM-1011 TEM, JEOL United States Inc., Peabody, MA). The image represents one of three independent experiments. **(B)** Venn Diagram analysis revealed that mice at 4 months old shared 16,079 peptides in their urinary exosomes with mice at 2 months old, while having 1,866 unique peptides. Conversely, mice at 2 months old also possessed 1,979 unique peptides. **(C)** A Volcano plot of 164 proteins increased and 176 protein decreased when SCD mice reached 4 month old compared with 2 month old. FC, fold change. **(D)**. Western blot analysis of protein abundance of heparanase (HPSE), cathepsin C (CATC), α2-macroglobulin (A2M), integrin αV (ITAV) and heat shock protein 27 (HSP27) in the urinary exosomes of non SCD (heterozygote) and SCD mice.

We extracted exosomes from 2 to 4 month old SCD mouse urine samples and confirmed their presence by electron microscopy (Figure 3A). We identified 19,924 peptides corresponding to 9,497 proteins, of which 1,979 peptides were specific to 2-month-old samples and 1,866 peptides were specific to 4-month-old samples, as shown by Venn diagram analysis (Figure 3B). The complete data was deposited into jPOST repository at the address https://repository.jpostdb.org/ with accession number PXD043401 and project number JPST002221. Label-free quantitation based on unique peptides ≥1 found in all 5 samples and 3 replicates in both groups revealed that 340 proteins were differentially secreted in the exosomes (164 increased and 176 decreased, *p* < 0.05, Figure 3C; Supplementary Table S1).

### Short-listed 9 proteins for further analyses

We focused on the 164 significantly increased proteins, as they are easier to monitor experimentally and potentially clinically than the decreased proteins. Based on the relevance to SCD pathophysiology, kidney injury and chronic kidney disease, we shortlisted 9 proteins for further evaluation (Table 2). Western blot analysis showed that excretion of heparanase, cathepsin C, α2-macroglobulin, and integrin αV was increased in SCD mice when they were 4 months old compared with a non-SCD mouse at the same age, while HSP27 was not detectable in their urine exosomes (Figure 3D). We could not identify a reliable signal with antibodies we have against other 5 proteins.

**TABLE 2:** Proteins shortlisted for further evaluation.

Proteins reported in the literature	Disease	References	Proteins identified in our proteomic analyses
Integrin α4β1	SCD	Lee et al. (2001)	Integrin αV
α2- macroglobulin	SCD	Makis et al. (2000)	α2-macroglobulin
iNOS	Sepsis-induced AKI	Heemskerk et al. (2006)	iNOS
Xanthine oxidase	Urinary tract infection	Ciragil et al. (2014)	Xanthine oxidase
Heparanase	Proteinuria in renal transplant patients	Shafat et al. (2012)	Heparanase
Cathepsins	SCD	Selma et al. (2022)	Cathepsin c
Collectin-12	Diabetic nephropathy	Caseiro et al. (2014)	Collectin-12
SERCA	Ischemia/reperfusion-induced heart injury	Tan et al. (2020)	SERCA3
Myosin-3	Radiation-induced nephropathy	Sharma et al. (2008)	Myosin-3

**FIGURE 4:**
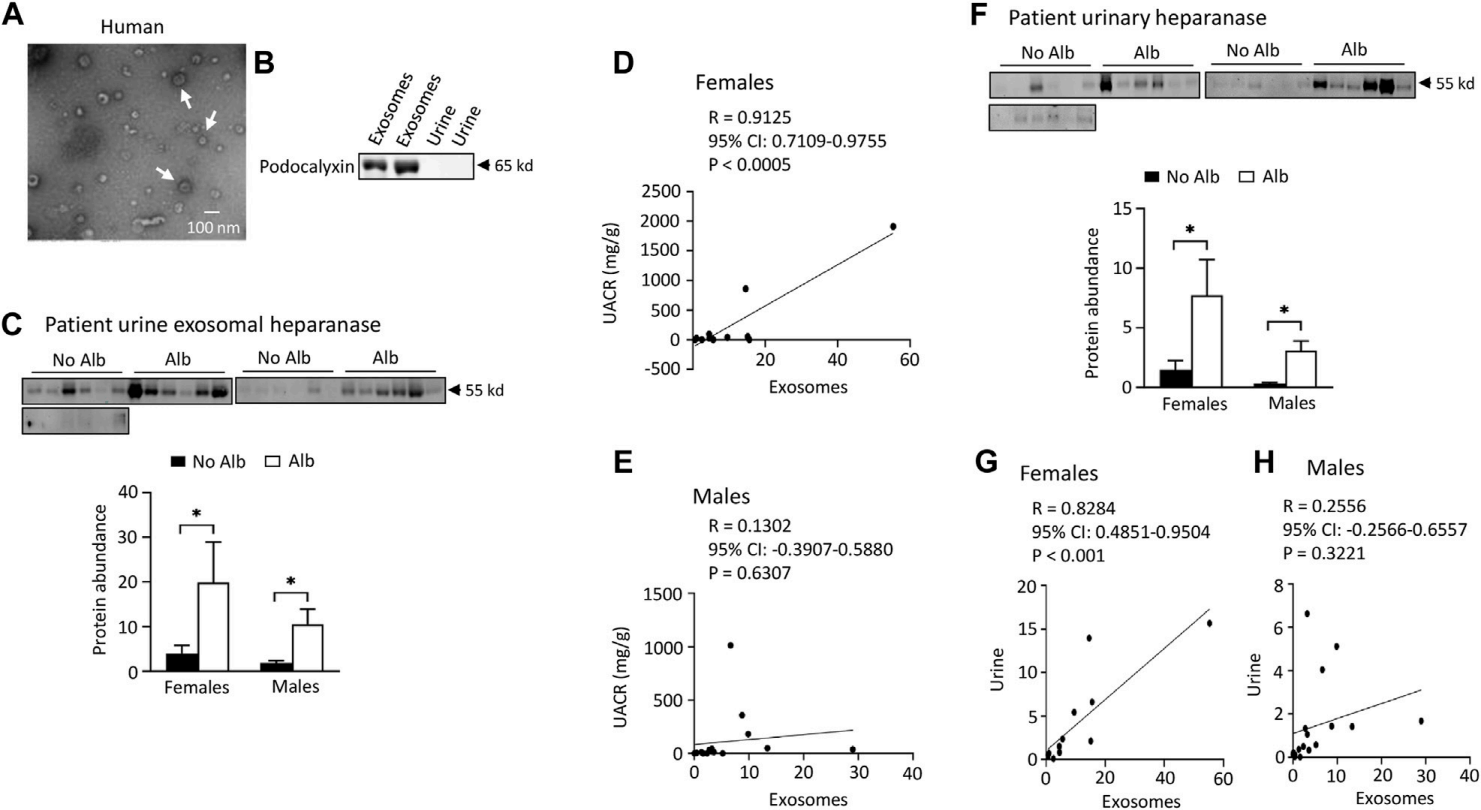
Elevated excretion levels of heparanase protein in urinary exosomes and urine are positively associated with albuminuria (Alb) in both male and female SCD subjects. However, only female subjects demonstrate stronger correlations between the excretion of urine exosomal heparanase and urine albumin creatinine ratio (UACR) and in excretion of heparanase between urinary exosomes and urine compared to male subjects. **(A)** Conformation of extraction of urinary exosomes by electron microscopy, a representative of three independent experiments. Urinary exosomes extraction and electron microscopic analysis were performed as described in Figure 3A. **(B)** Conformation of extraction of urinary exosomes by Western blot analysis. Each lane was loaded with 30 µg proteins. **(C)** Both male and female SCD subjects increased excretion of heparanase in their urine exosomes upon developing albuminuria as determined by Western blot analyses (Males: *n* = 10 for No Alb, *n* = 7 for Alb, Females: *n* = 8 for No Alb, *n* = 5 for Alb). **(D,E)** Increased excretion of heparanase in the urinary exosomes correlated with UACR only in female subjects, but not in male subjects (Pearson test, Males: *n* = 9 for No Alb, *n* = 7 for Alb, Females: *n* = 7 for No Alb, *n* = 5 for Alb). **(F)** Western blot analyses indicate that both male and female SCD subjects increased excretion of heparanase protein in their urine upon developing albuminuria (Males: *n* = 10 for No Alb, *n* = 7 for Alb, Females: *n* = 8 for No Alb, *n* = 5 for Alb). **(G,H)** Only female subjects demonstrated a good correlation in excretion of heparinase protein between urinary exosomes and urine (Pearson test, Males: *n* = 9 for No Alb, *n* = 7 for Alb, Females: *n* = 7 for No Alb, *n* = 5 for Alb). (*p < 0.05, two-tailed *t*-test).

*Both male and female subjects increase or have a tendency to increase the excretion of heparanase, cathepsin C, α2-macroglobulin, and SERCA3 in their urinary exosomes upon developing albuminuria, but female subjects demonstrate stronger correlations between the excretion of these proteins and urine albumin creatinine ratio (UACR) compared to male subjects*. Our ultimate objective is to identify a biomarker capable of diagnosing sickle nephropathy before the onset of albuminuria. To explore potential candidates, we investigated whether our findings from mouse urine exosomes could be replicated in the urinary exosomes of patients. Successful validation in humans would allow us to design a time-course study to assess whether these protein levels increase before the onset of albuminuria in patients. Therefore, we analyzed 18 subjects without albuminuria and 8 subjects with microalbuminuria and 4 participants with macroalbuminuria (Table 1). Among the participants, 54% were male and 97% were Black or African American (Table 1). We confirmed the isolation of exosomes with electron microscopy (Figure 4A) and Western blot analysis with an antibody against podocalyxin, a biomarker of exosomes (Figure 4B). Both male and female SCD subjects with albuminuria exhibited an increase in or a tendency to increase excretion of heparanase, cathepsin C, α2-macroglobulin, and SERCA3 in their urinary exosomes compared with subjects without albuminuria (Figures 4C-H to Figure 7). However, female subjects had better correlations of protein excretion with UACR than male subjects with Pearson correlation coefficient value 0.9125, 0.8222, 0.7813 and 0.9791, respectively. In contrast, the Pearson correlation coefficient value for males were 0.1302, 0.7022, −0.03842 and 0.0691, respectively (Figures 4C-H to Figure 7). Neither female nor male subjects showed a correlation between excretion of these proteins in the exosomes and eGFR CKD-EPI Creatinine Equation (2021) (Data not shown). We were unable to obtain a reliable signal using the antibody we have against human integrin αV.

**FIGURE 5:**
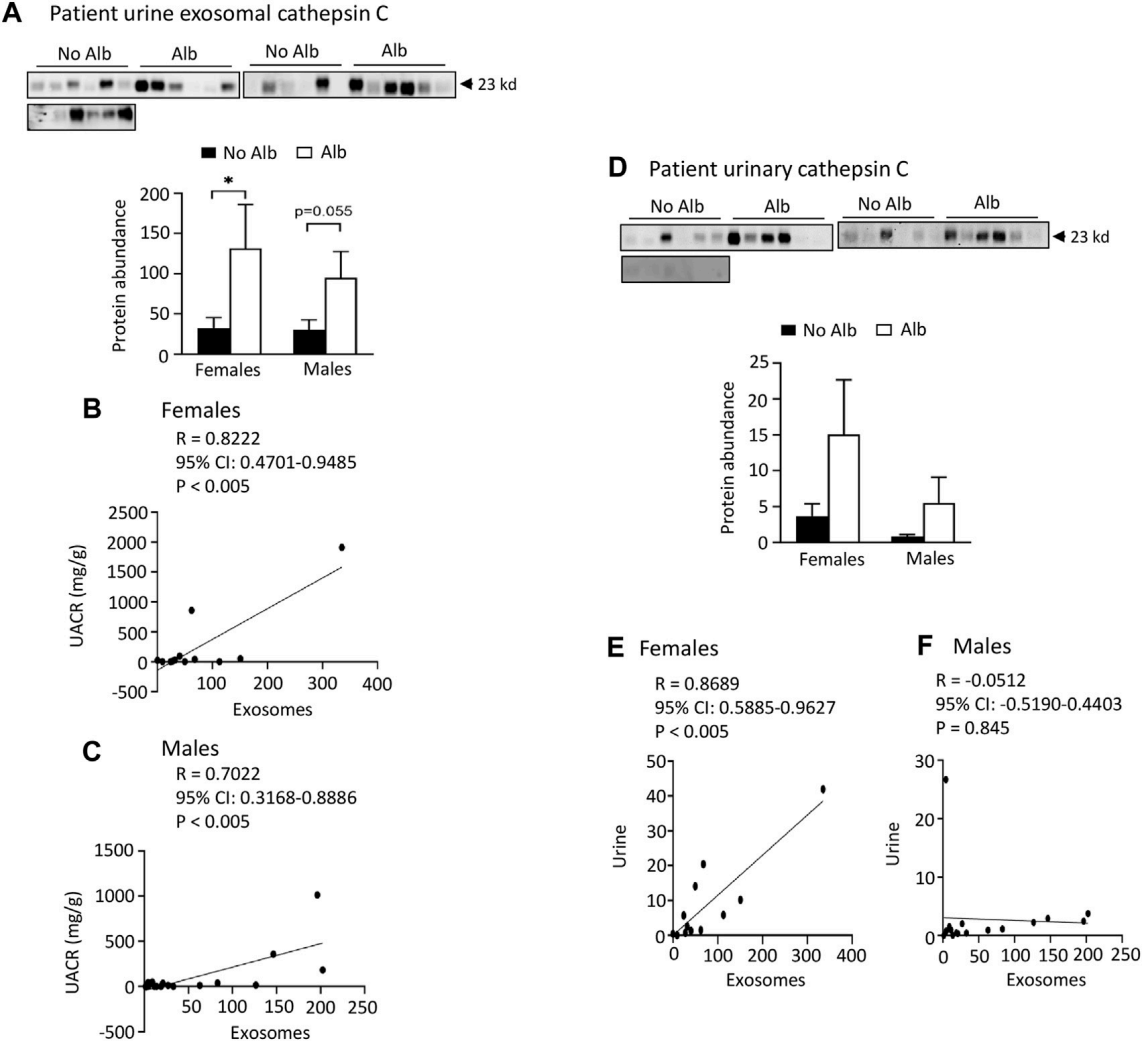
Elevated levels of cathepsin C protein excretion in both urinary exosomes and urine demonstrate a positive association with albuminuria (Alb) in male and female SCD subjects. Importantly, the correlations between urine exosomal cathepsin C excretion and the urine albumin creatinine ratio (UACR), as well as cathepsin C excretion between urinary exosomes and urine, are particularly pronounced in female subjects compared to their male counterparts. **(A)** Western blot analyses of cathepsin C in the urinary exosomes (Males: *n* = 10 for No Alb, *n* = 7 for Alb, Females: *n* = 8 for No Alb, *n* = 5 for Alb). **(B,C)** The elevated excretion of cathepsin C in urinary exosomes exhibited a stronger correlation with urine UACR in female subjects compared to male subjects (Pearson test, Males: *n* = 9 for No Alb, *n* = 7 for Alb, Females: *n* = 7 for No Alb, *n* = 5 for Alb). **(D)** Both male and female subjects with albuminuria tended to increase urinary excretion of cathepsin C as shown by Western blot analyses (Males: *n* = 10 for No Alb, *n* = 7 for Alb, Females: *n* = 8 for No Alb, *n* = 5 for Alb). **(E,F)** Increased excretion of cathepsin C in the urinary exosomes correlated with its excretion in urine in female subjects, but not in male subjects (Pearson test, Males: *n* = 9 for No Alb, *n* = 7 for Alb, Females: *n* = 7 for No Alb, *n* = 5 for Alb). (*p < 0.05, two-tailed *t*-test).

**FIGURE 6:**
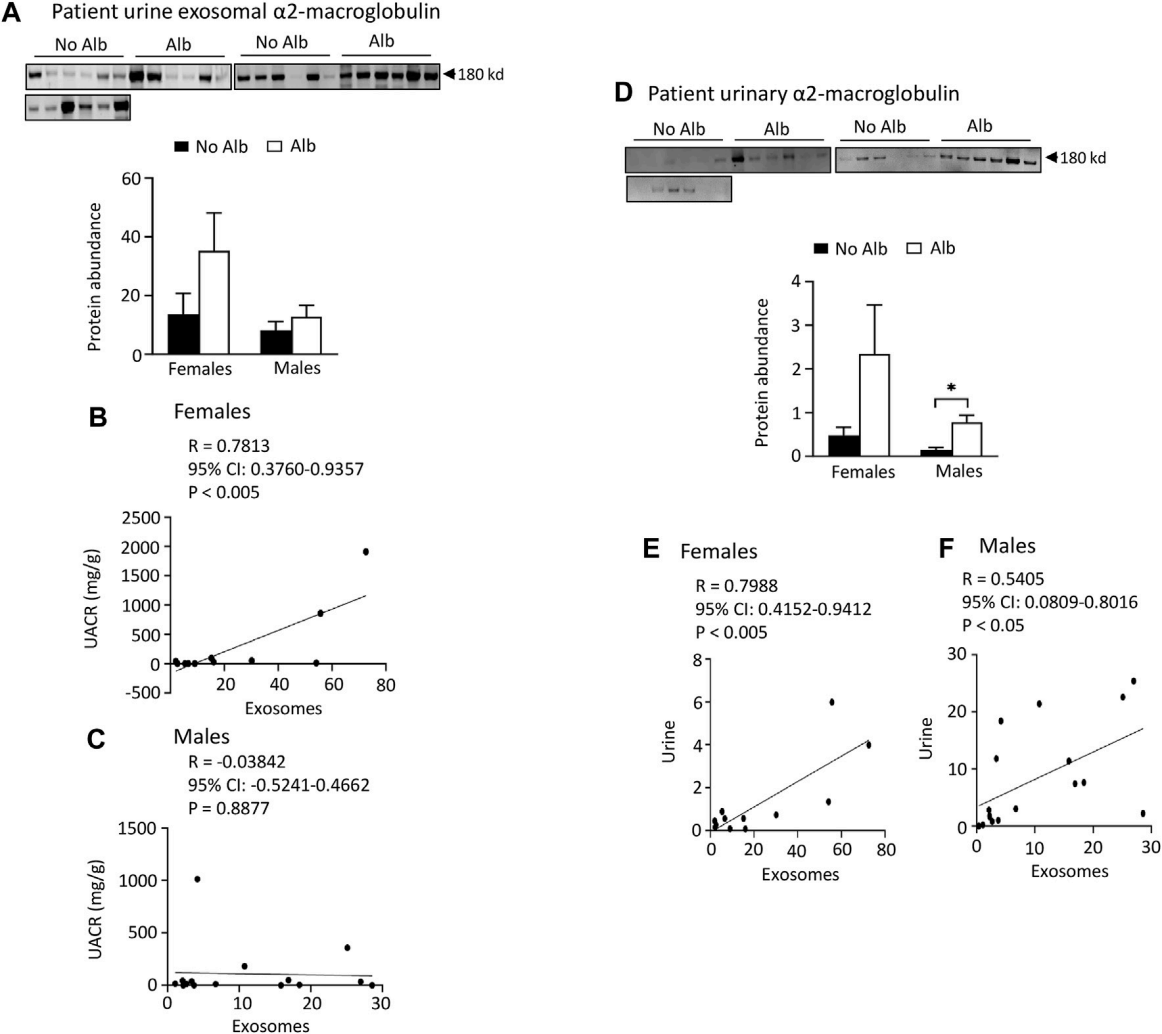
Excretion of α2-macroglobulin protein in both urinary exosomes and urine is increased in parallel with albuminuria (Alb) in male and female SCD subjects. However, female subjects exhibit better correlations between urine exosomal α2-macroglobulin excretion and the urine albumin creatinine ratio (UACR), as well as in α2-macroglobulin excretion between urinary exosomes and urine, compared to their male counterparts. **(A)** Western blot analyses of α2-macroglobulin protein in the urinary exosomes (Males: *n* = 10 for No Alb, *n* = 7 for Alb, Females: *n* = 8 for No Alb, *n* = 5 for Alb). **(B,C)** Only female subjects, but not male subjects, displayed a correlation in excretion of α2-macroglobulin in the urinary exosomes with UACR (Pearson test, Males: *n* = 9 for No Alb, *n* = 7 for Alb, Females: *n* = 7 for No Alb, *n* = 5 for Alb). **(D)** Western blot analyses of α2-macroglobulin protein in both male and female subjects’ urine (Males: *n* = 10 for No Alb, *n* = 7 for Alb, Females: *n* = 8 for No Alb, *n* = 5 for Alb). **(E,F)** Increased excretion of α2-macroglobulin protein in the urinary exosomes correlated better with its excretion in urine in female subjects than in male subjects (Pearson test, Males: *n* = 9 for No Alb, *n* = 7 for Alb, Females: *n* = 7 for No Alb, *n* = 5 for Alb). (*p < 0.05, two-tailed *t*-test).

*Both male and female SCD subjects with albuminuria exhibit significantly increased or tend to excrete higher levels of heparanase, cathepsin C, and α2-macroglobulin in their urine compared to those without albuminuria, but only female subjects demonstrated superior correlations between the excretion of these proteins in urinary exosomes and urine, in contrast to male subjects.* To expedite detection, we explored whether heparanase, cathepsin C, α2-macroglobulin, and SERCA3 could be directly identified in participants’ urine using Western blot analysis. In the presence of albuminuria, both male and female SCD subjects excreted significantly higher levels or exhibited a tendency to excrete more heparanase, cathepsin C, and α2-macroglobulin in their urine compared to subjects without albuminuria (Figures 4C-H to Figure 6). However, only female subjects exhibited a robust correlation of protein excretion in urine with urinary exosomes, with Pearson correlation coefficient values of 0.8284, 0.8689, and 0.7988, respectively, while the corresponding values for males were only 0.2556, −0.0512, and 0.5405 (Figures 4C-H to Figure 6). Unfortunately, the urine SERCA3 signal was too low to be considered reliable.

**FIGURE 7:**
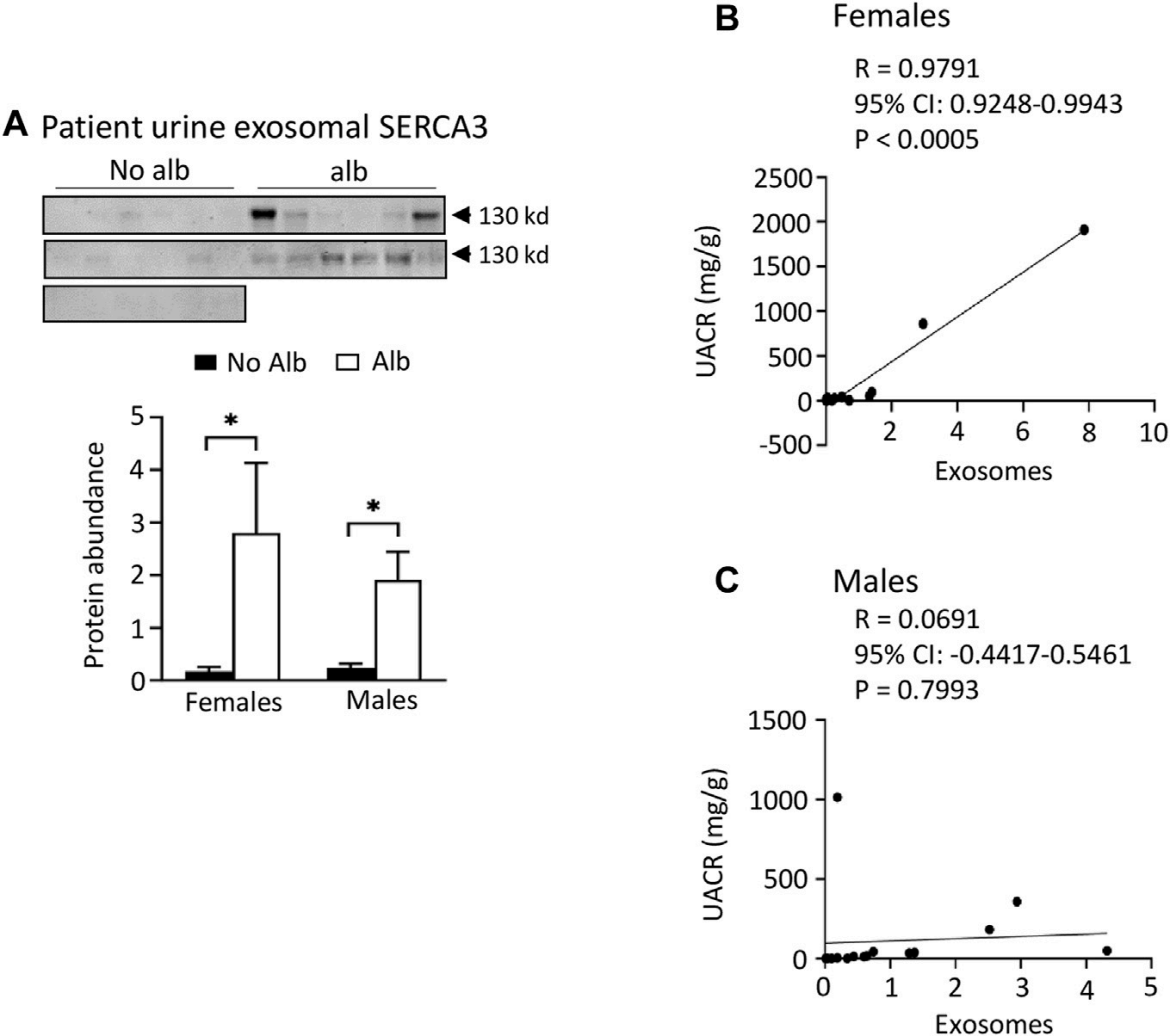
Both male and female SCD subjects with albuminuria increases excretion of SERCA3 protein in the urinary exosomes. However, only female subjects show a correlation of urine exosomal excretion of SERCA3 protein with the urine albumin creatinine ratio (UACR), whereas male subjects do not. **(A)** Western blot analyses of SERCA3 in the urinary exosomes (Males: *n* = 10 for No Alb, *n* = 7 for Alb, Females: *n* = 8 for No Alb, *n* = 5 for Alb). **(B,C)** A strong correlation between the urine exosomal excretion of SERCA3 protein and UACR was observed only in female subjects, with no such correlation found in male subjects (Pearson test, Males: *n* = 9 for No Alb, *n* = 7 for Alb, Females: *n* = 7 for No Alb, *n* = 5 for Alb). (*p < 0.05, two-tailed *t*-test).

**FIGURE 8:**
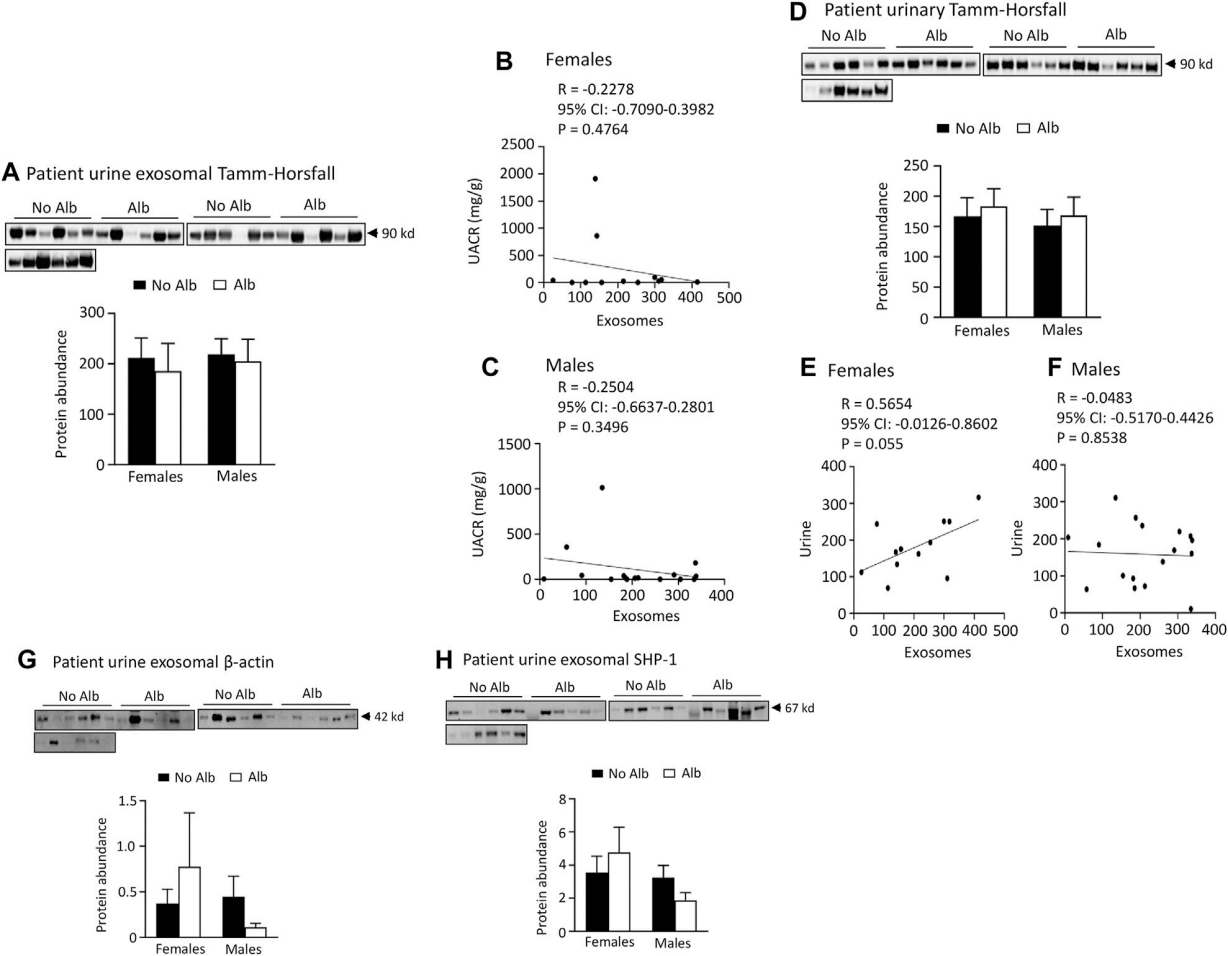
There is no association of excretion of Tamm-Horsfall protein (THP), β-actin, or SHP-1 protein in urinary exosomes with albuminuria in either male or female subjects. There is no association between excretion of THP and urine albumin creatinine ratio (UACR) in either male or female subjects. Only female subjects display a weak correlation in excretion of THP between urinary exosomes and urine. **(A)** Western blot analyses of THP in urinary exosomes (Males: *n* = 10 for No Alb, *n* = 7 for Alb, Females: *n* = 8 for No Alb, *n* = 5 for Alb). **(B,C)** No correlation was observed between the excretion of THP in urinary exosomes and UACR in either sex (Pearson test, Males: *n* = 9 for No Alb, *n* = 7 for Alb, Females: *n* = 7 for No Alb, *n* = 5 for Alb). **(D)** There is no association of urinary excretion of THP with albuminuria in either male or female subjects as examined by Western blot analysis (Males: *n* = 10 for No Alb, *n* = 7 for Alb, Females: *n* = 8 for No Alb, *n* = 5 for Alb). **(E,F)** A weak correlation in the excretion of THP between urinary exosomes and urine was found only in female subjects (Pearson test, Males: *n* = 10 for No Alb, *n* = 7 for Alb, Females: *n* = 7 for No Alb, *n* = 5 for Alb). **(G,H)** No correlation was found in the excretion of β-actin or SHP-1 protein in urinary exosomes with albuminuria in either sex (Males: *n* = 10 for No Alb, *n* = 7 for Alb, Females: *n* = 8 for No Alb, *n* = 5 for Alb).

*Neither male nor female subjects significantly increased the excretion of Tamm-Horsfall protein (THP), β-actin, or SH2-domain-containing protein tyrosine phosphatase-1 (SHP-1) in their urinary exosomes upon developing albuminuria*. To examine whether the increased excretion of heparanase, cathepsin C, α2-macroglobulin and SERCA3 in SCD subjects’ urinary exosomes specifically correlated with albuminuria, we examined the excretion of THP in urine exosomes and urine. The excretion of THP in both urinary exosomes and urine was unaffected by albuminuria in male or female subjects, and there was no correlation between their excretion levels in urinary exosomes and UACR with the Pearson correlation coefficient value only −0.2504 and −0.2278, respectively (Figure 8). There was a weak correlation in excretion of THP protein between urinary exosomes and urine in female subjects only, but not in male subjects with the Pearson correlation coefficient value 0.5654 and −0.0483, respectively (Figure 8). THP is almost exclusively produced by the epithelial cells lining the thick ascending limb of the loop of Henle and early distal tubules, and it does not represent the glomeruli or other segments of the nephron (Micanovic et al., 2020). Therefore, we examined the excretion of β-actin, a universally expressed protein, and SHP-1, which is expressed in the kidney cortex, outer and inner medullas (Wang et al., 2015), in the exosomes. The excretion of these two proteins in the exosomes is independent of albuminuria as well in male or female subjects (Figure 8). These findings indicate that the increased excretion of heparanase, cathepsin C, α2-macroglobulin and SERCA3 in SCD subjects’ urinary exosomes specifically correlated with albuminuria.

## Discussion

Exosomes are small extracellular vesicles, typically ranging from 30 to 150 nm in diameter, known for their role in intercellular communication. Urinary exosomes, originating primarily from renal epithelial cells and podocytes, provide a rich source of cellular information, emerging as promising candidates for biomarker discovery in kidney diseases. The unbiased nature of proteomic analysis allows for a comprehensive evaluation of sample contents, which is crucial in identifying potential biomarkers. In this regard, proteomic analyses of urinary exosomes have been explored for identification of potential biomarkers for early diagnosis and monitoring of various renal diseases, including acute kidney and podocyte injuries (Zhou et al., 2006b; Zhou et al. 2008; Zhou et al. 2013; Awdishu et al., 2021), glomerular kidney disease (Gutwein et al., 2010), kidney transplant rejection (Kim et al., 2022), and diabetic nephropathy (Liu et al., 2022).

With a similar approach, we observed an increase in the excretion of 164 proteins and a decrease in 176 proteins in the exosomes of mice with albuminuria compared to without. These findings were partly validated through Western blot analysis. Extending this investigation to SCD subjects, we noted a significant increase or an upward trend in the excretion of heparanase, cathepsin C, α2-macroglobulin, and SERCA3 proteins in urinary exosomes of both male and female SCD patients with albuminuria. However, female subjects demonstrated stronger correlations between these protein excretions and UACR than male subjects. Furthermore, the correlations between the excretion of heparanase, cathepsin C, and α2-macroglobulin in urinary exosomes and urine were more pronounced in female subjects compared to male subjects. In contrast, no association was found between albuminuria and the excretion of Tamm-Horsfall protein, β-actin, or SHP-1 protein in urinary exosomes of either sex. These findings suggest that the increased excretion of heparanase, cathepsin C, α2-macroglobulin, and SERCA3 proteins from urinary exosomes or urine specifically correlates with albuminuria. The stronger correlations observed in female participants suggest a sex-specific response in the pathophysiology of SCN, possibly due to sex-specific mechanisms influencing the activity or excretion of these proteins in SCD.

The specificity of increased excretion of heparanase, cathepsin C, α2-macroglobulin, and SERCA3 proteins in urinary exosomes for SCN, as opposed to general proteinuria, warrants further investigation. In SCD, coagulation is activated, with hypercoagulability being a contributing factor to SCN (Nasimuzzaman and Malik, 2019). Heparanase plays a key role in coagulation by upregulating tissue factor (Abassi and Goligorsky, 2020), while α2-macroglobulin acts as a fibrinolysis inhibitor by targeting plasmin and thrombin (Lagrange et al., 2022). Early research indicated no significant difference in serum α2-macroglobulin levels between SCD patients and healthy controls (Hedo et al., 1993), but later findings showed elevated α2-macroglobulin in steady-state SCD patients compared to healthy subjects (Makis et al., 2000).

However, the involvement of heparanase and α2-macroglobulin in other renal diseases leading to proteinuria has been documented. Heparanase has been implicated in various experimental and human glomerular diseases associated with proteinuria, including diabetes and membranous nephropathy (Shafat et al., 2011; Gil et al., 2012; Szymczak et al., 2017). Elevated urinary protein levels and activity of heparanase are noted in patients with both Type 1 and Type 2 diabetes (Shafat, Ilan, Zoabi, Vlodavsky and Nakhoul, 2011; Rops et al., 2012), as well as in renal transplant patients with proteinuria and decreased allograft function (Shafat et al., 2012). Similarly, increased serum α2-macroglobulin levels, correlating with microalbuminuria, have been observed in diabetic patients (James et al., 1980; Ahmad et al., 2001; Yoshino et al., 2019). The roles of cathepsin C and SERCA3 in SCD, and particularly in SCN, remain largely unexplored. Nonetheless, by combining patients’ history with other diagnostic tools, SCN can be differentiated from other types of disease-induced nephropathy using these potential biomarkers.

It is noteworthy that GFR, measured using a transcutaneous method, was found to be elevated in SCD mice. This observation mirrors the renal function in patients with SCD during the early stages of the disease. The potential underlying causes for this include compensatory high renal blood flow and hyperfiltration, endothelial dysfunction, altered nitric oxide metabolism, and changes in hormonal and cytokine levels (Nath and Hebbel, 2015; Kasztan et al., 2019; Afangbedji and Jerebtsova, 2022).

In summary, through proteomic analyses of urinary exosomes from humanized SCD mice, and subsequent Western blot confirmation using urine and urinary exosomes of SCD subjects, we have observed that both male and female SCD subjects exhibited an increased or trending increase in the excretion of heparanase, cathepsin C, α2-macroglobulin, and SERCA3 proteins. Notably, this increase was specifically correlated with albuminuria. Moreover, we have found that female subjects demonstrated stronger correlations between the excretion of these proteins and the UACR compared to their male counterparts. One limitation of the present study is the small size of the human subject population. Despite this, the study lays a solid foundation for further exploration into the potential use of these proteins as biomarkers for the early diagnosis of SCN and for monitoring therapeutic efficacy, particularly in female patients. The next phase of our investigation will involve using a different cohort to validate these discoveries. Subsequently, a prospective, observational study will be conducted to determine whether these protein levels increase prior to the onset of albuminuria in SCD patients’ urine and urinary exosomes.

## Data Availability

The data presented in the study are deposited in the jPOST repository at the address https://repository.jpostdb.org/ with accession number PXD043401 and project number JPST002221.

## Abbreviations

A2M, α2-macroglobulin; CATC, cathepsin C; H & E, hematoxylin-eosin; FITC, fluorescein isothiocyanate; GFR, glomerular filtration rate; eGFR, estimated glomerular filtration rate; HPSE, heparanase; KIM-1, kidney injury molecule-1; LC-MS/MS, liquid chromatography-tandem mass spectrometry; MTC, Masson’s trichrome; SCD, sickle cell disease; SCN, sickle cell disease-induced nephropathy; SERCA3, sarcoplasmic endoplasmic Ca^2+^ ATPase-3; SHP-1, SH2-domain-containing protein tyrosine phosphatase-1; THP, Tamm-Horsfall protein; tGFR, transcutaneous glomerular filtration rate; UACR, urine albumin creatinine ratio.
